# Cannabidiol‐Mediated Neuroprotection in Aβ42‐Induced Alzheimer's Model of Drosophila: Behavioral and Morphological Evidence

**DOI:** 10.1002/alz70856_096378

**Published:** 2025-12-24

**Authors:** Abbas Nasidi, Uduak Emmanuel Umana, Adamu Abubakar Sadeeq, Musa Mustapha

**Affiliations:** ^1^ Bauchi State University, Gadau, Bauchi, Nigeria; ^2^ Ahmadu Bello University, Zaria, Kaduna, Nigeria

## Abstract

**Background:**

Alzheimer's disease (AD) is a progressive and irreversible neurodegenerative disorder characterized by cognitive decline and neuropathological transformations, imposing a significant burden on individuals and healthcare systems globally. Despite ongoing research endeavors, effective treatments to halt AD progression remain elusive. Cannabidiol (CBD) is a natural compound derived from cannabis renowned for its anti‐inflammatory, neuroprotective, and antioxidant properties. This study investigated the neuroprotective potential of CBD in mediating neurobehavioral and morphological changes in the Aβ42 transgenic model of AD.

**Method:**

150 flies were grouped into five. Group I & II are negative and positive control and were exposed to 10 g of diet only, group III is an experimental control and was exposed to 1 mM Donepezil. Group IV & V were subjected to 2 mg and 4 mg of CBD respectively for 2 weeks. Motor function, memory abilities, social interactions, and expression of amyloid beta (Aβ42) and glial fibrillary acidic protein (GFAP) were evaluated using climbing, aversive phototaxis suppression, social space assay, and immunostaining respectively.

**Result:**

Findings revealed a significant decrease in motor coordination (0.31 ± 0.08, *p* = 0.007), memory function (7.00 ± 8.52, *p* = 0.008), and social behavior (3.09 ± 0.51, *p* = 0.0008) in the positive control compared to the negative control group, accompanied by elevated Aβ42 and GFAP expression (58.50 ± 8.000, *p* = 0.03). However, treatment with CBD effectively mitigated these deficits. Motor function was restored in the 4 mg CBD (0.69 ± 0.08, *p* = 0.028), memory abilities were improved in the 4 mg CBD (63.00 ± 7.35, *p* = 0.007), social interaction was increased in the 4 mg CBD group (1.19 ± 0.53, *p* = 0.0071). Furthermore, CBD treatment reduced Aβ42 and GFAP immunoreactivity (58.50 ± 8.000, *p* = 0.03).

**Conclusion:**

This study provides compelling evidence for the therapeutic potential of CBD oil in mitigating motor and cognitive deficits and neuropathological changes associated with AD, underscoring the importance of further research into the mechanisms of action and optimization of treatment regimens for AD.

